# Supplementary material for: Probiotic as a Potential Gut Microbiome Modifier for Stroke Treatment: A Systematic Scoping Review of In Vitro and In Vivo Studies
Source: Nutrients. 2022 Sep 5;14(17):3661. doi: 10.3390/nu14173661 (PMC9460291; doi:10.3390/nu14173661)
Supplement: Supplementary file 1 [file nutrients-14-03661-s001.zip › nutrients-1855512-supplementary.pdf]

Table S1. Probiotics search term

Set #	Pubmed	Results
#1 Probiotics	"probiotics"[MeSH Terms] OR probiotic*[tiab] OR “prebiotics”[MeSH Terms] OR prebiotics*[tiab] OR "synbiotics"[MeSH Terms] OR synbiotic*[tiab] OR "lactobacillales"[MeSH Terms] OR “lactobacillus”[MeSH Terms] OR lactobacill*[tiab] OR pediococcus[tiab] OR enterococcus[tiab] OR leuconostoc[tiab] OR oenococcus[tiab] OR weissella[tiab] OR lactococcus[tiab] OR streptococcus[tiab] OR "bifidobacterium"[MeSH Terms] OR bifidobacteri*[tiab] OR aeriscardovia[tiab] OR alloscardovia[tiab] OR bombiscardovia[tiab] OR galliscardovia[tiab] OR neoscardovia[tiab] OR parascardovia[tiab] OR pseudoscardovia[tiab] OR scardovia[tiab] OR "gardnerella"[MeSH Terms] OR gardnerella[tiab] OR "propionibacterium"[MeSH Terms] OR propionibacteri*[tiab] OR "staphylococcus"[MeSH Terms] OR staphylococcus[tiab] OR "bacillales"[MeSH Terms] OR bacillus[tiab] OR bacill*[tiab] OR "saccharomyces"[MeSH Terms] OR saccharomyces[tiab] OR “L. acidophilus”[tiab] OR “L. brevis”[tiab] OR “L. casei”[tiab] OR “L.delbrueckii”[tiab] OR “L. fermentum”[tiab] OR “L. gasseri”[tiab] OR “L. helveticus”[tiab] OR “L. johnsonii”[tiab] OR “L. reuteri”[tiab] OR “L. rhamnosus”[tiab] OR “L. salivarius”[tiab] OR “L. sporogenes”[tiab] OR “B. animalis”[tiab] OR “B. bifidum”[tiab] OR “B. breve”[tiab] OR “B. longum”[tiab] OR “E. faecium”[tiab]	**613014**
#2 Stroke	“ischemic stroke”[MeSH Terms] OR “cerebrovascular disorders”[MeSH Terms:noexp] OR “basal ganglia cerebrovascular disease”[MeSH Terms] OR “brain ischemia”[MeSH Terms] OR “carotid artery diseases”[MeSH Terms] OR “cerebral small vessel diseases”[MeSH Terms] OR “stroke, lacunar”[MeSH Terms] OR “intracranial arterial diseases”[MeSH Terms:noexp] OR “cerebral arterial diseases”[MeSH Terms] OR “intracranial arteriosclerosis”[MeSH Terms] OR “intracranial embolism and thrombosis”[MeSH Terms] OR “vasospasm, intracranial”[MeSH Terms] OR stroke*[tiab] OR cerebrovasc*[tiab] OR CVA[tiab] OR ESUS[tiab] OR TIA[tiab] OR (transient[tiab] AND (ischemic[tiab] OR ischaemic[tiab]) AND attack[tiab]) OR ((cerebr*[tiab] OR brain[tiab] OR cerebell*[tiab] OR arteriovenous[tiab] OR intracran*[tiab] OR intracerebr*[tiab] OR intracerebel*[tiab] OR MCA[tiab] OR ACA[tiab] OR PCA[tiab] OR “basal ganglia”[tiab] OR lacunar[tiab] OR cortical[tiab] OR carotid[tiab]) AND (ischemi*[tiab] OR ischaemi*[tiab] OR infarct*[tiab] OR injur*[tiab] OR accident*[tiab] OR thrombo*[tiab] OR emboli*[tiab] OR hypoxi*[tiab] OR occlus*[tiab] OR obstruct*[tiab] OR vasospasm[tiab] OR vasoconstrict*[tiab])) OR “cardioembolism”[tiab] OR moyamoya[tiab] OR “moya moya”[tiab]	**660563**
#3	#1 AND #2	**2226**

Set #	Embase	Results
#1<br>Probiotics	'probiotic agent'/exp OR 'probiotic*':ti,ab OR 'prebiotic agent'/exp OR 'prebiotic*':ti,ab OR 'synbiotic agent'/exp OR 'synbiotic*':ti,ab OR 'lactobacillales'/exp OR 'lactobacillus'/exp OR 'lactobacill*':ti,ab OR 'pediococcus':ti,ab OR 'enterococcus':ti,ab OR 'leuconostoc':ti,ab OR 'oenococcus':ti,ab OR 'weissella':ti,ab OR 'lactococcus':ti,ab OR 'streptococcus':ti,ab OR 'bifidobacterium'/exp OR 'bifidobacteri*':ti,ab OR 'aeriscardovia':ti,ab OR 'alloscardovia':ti,ab OR 'bombiscardovia':ti,ab OR 'galliscardovia':ti,ab OR 'neoscardovia':ti,ab OR 'parascardovia':ti,ab OR 'pseudoscardovia':ti,ab OR 'scardovia':ti,ab OR 'gardnerella'/exp OR 'gardnerella':ti,ab OR 'propionibacterium'/exp OR 'propionibacteri*':ti,ab OR 'staphylococcus'/exp OR 'staphylococcus':ti,ab OR 'bacillales'/exp OR 'bacillus':ti,ab OR 'bacill*':ti,ab OR 'saccharomyces'/exp OR 'saccharomyces':ti,ab OR 'L. acidophilus':ti,ab OR 'L. brevis':ti,ab OR 'L. casei':ti,ab OR 'L.delbrueckii':ti,ab OR 'L. fermentum':ti,ab OR 'L. gasseri':ti,ab OR 'L. helveticus':ti,ab OR 'L. johnsonii':ti,ab OR 'L. reuteri':ti,ab OR 'L. rhamnosus':ti,ab OR 'L. salivarius':ti,ab OR 'L. sporogenes':ti,ab OR 'B. animalis':ti,ab OR 'B. bifidum':ti,ab OR 'B. breve':ti,ab OR 'B. longum':ti,ab OR 'E. faecium':ti,ab	**781596**
#2<br>Stroke	'brain ischemia'/exp OR 'cerebrovascular disease'/de OR 'brain infarction'/exp OR 'carotid artery disease'/exp OR 'cerebral artery disease'/exp OR 'lacunar stroke'/exp OR 'cardioembolic stroke'/exp OR 'brain atherosclerosis'/exp OR 'occlusive cerebrovascular disease'/exp OR 'brain vasospasm'/exp OR 'stroke*':ti,ab OR 'cerebrovasc*':ti,ab OR 'CVA':ti,ab OR 'ESUS':ti,ab OR 'TIA':ti,ab OR ('transient' NEAR/3 ('ischemic' OR 'ischaemic') NEAR/3 'attack'):ti,ab OR (('cerebr*' OR 'brain' OR 'cerebell*' OR 'arteriovenous' OR 'intracran*' OR 'intracerebr*' OR 'intracerebel*' OR 'MCA' OR 'ACA' OR 'PCA' OR 'basal ganglia' OR 'lacunar' OR 'cortical' OR 'carotid') NEAR/3 ('ischemi*' OR 'ischaemi*' OR 'infarct*' OR 'injur*' OR 'accident*' OR 'thrombo*' OR 'emboli*' OR 'hypoxi*' OR 'occlus*' OR 'obstruct*' OR 'vasospasm' OR 'vasoconstrict*')):ti,ab OR 'cardioembolism':ti,ab OR 'moyamoya':ti,ab OR 'moya moya':ti,ab	**818655**
#3	#1 AND #2	**3926**

Set #	Central	Results
#1 Probiotics	[mh "probiotics"] OR probiotic*:ti,ab,kw OR [mh “prebiotics”] OR prebiotics*:ti,ab,kw OR [mh "synbiotics"] OR synbiotic*:ti,ab,kw OR [mh "lactobacillales"] OR [mh “lactobacillus”] OR lactobacill*:ti,ab,kw OR pediococcus:ti,ab,kw OR enterococcus:ti,ab,kw OR leuconostoc:ti,ab,kw OR oenococcus:ti,ab,kw OR weissella:ti,ab,kw OR lactococcus:ti,ab,kw OR streptococcus:ti,ab,kw OR [mh "bifidobacterium"] OR bifidobacteri*:ti,ab,kw OR aeriscardovia:ti,ab,kw OR alloscardovia:ti,ab,kw OR bombiscardovia:ti,ab,kw OR galliscardovia:ti,ab,kw OR neoscardovia:ti,ab,kw OR parascardovia:ti,ab,kw OR pseudoscardovia:ti,ab,kw OR scardovia:ti,ab,kw OR [mh "gardnerella"] OR gardnerella:ti,ab,kw OR [mh "propionibacterium"] OR propionibacteri*:ti,ab,kw OR [mh "staphylococcus"] OR staphylococcus:ti,ab,kw OR [mh "bacillales"] OR bacillus:ti,ab,kw OR bacill*:ti,ab,kw OR [mh "saccharomyces"] OR saccharomyces:ti,ab,kw OR “L. acidophilus”:ti,ab,kw OR “L. brevis”:ti,ab,kw OR “L. casei”:ti,ab,kw OR “L.delbrueckii”:ti,ab,kw OR “L. fermentum”:ti,ab,kw OR “L. gasseri”:ti,ab,kw OR “L. helveticus”:ti,ab,kw OR “L. johnsonii”:ti,ab,kw OR “L. reuteri”:ti,ab,kw OR “L. rhamnosus”:ti,ab,kw OR “L. salivarius”:ti,ab,kw OR “L. sporogenes”:ti,ab,kw OR “B. animalis”:ti,ab,kw OR “B. bifidum”:ti,ab,kw OR “B. breve”:ti,ab,kw OR “B. longum”:ti,ab,kw OR “E. faecium”:ti,ab,kw	**21068**
#2 Stroke	[mh “ischemic stroke”] OR [mh ^“cerebrovascular disorders”] OR [mh “basal ganglia cerebrovascular disease”] OR [mh “brain ischemia”] OR [mh “carotid artery diseases”] OR [mh “cerebral small vessel diseases”] OR [mh “stroke, lacunar”] OR [mh ^“intracranial arterial diseases”] OR [mh “cerebral arterial diseases”] OR [mh “intracranial arteriosclerosis”] OR [mh “intracranial embolism and thrombosis”] OR [mh “vasospasm, intracranial”] OR stroke*:ti,ab,kw OR cerebrovasc*:ti,ab,kw OR CVA:ti,ab,kw OR ESUS:ti,ab,kw OR TIA:ti,ab,kw OR (transient:ti,ab,kw AND (ischemic:ti,ab,kw OR ischaemic:ti,ab,kw) AND attack:ti,ab,kw) OR ((cerebr*:ti,ab,kw OR brain:ti,ab,kw OR cerebell*:ti,ab,kw OR arteriovenous:ti,ab,kw OR intracran*:ti,ab,kw OR intracerebr*:ti,ab,kw OR intracerebel*:ti,ab,kw OR MCA:ti,ab,kw OR ACA:ti,ab,kw OR PCA:ti,ab,kw OR “basal ganglia”:ti,ab,kw OR lacunar:ti,ab,kw OR cortical:ti,ab,kw OR carotid:ti,ab,kw) AND (ischemi*:ti,ab,kw OR ischaemi*:ti,ab,kw OR infarct*:ti,ab,kw OR injur*:ti,ab,kw OR accident*:ti,ab,kw OR thrombo*:ti,ab,kw OR emboli*:ti,ab,kw OR hypoxi*:ti,ab,kw OR occlus*:ti,ab,kw OR obstruct*:ti,ab,kw OR vasospasm:ti,ab,kw OR vasoconstrict*:ti,ab,kw)) OR “cardioembolism”:ti,ab,kw OR moyamoya:ti,ab,kw OR “moya moya”:ti,ab,kw	**85342**
#3	#1 AND #2	**141**
